# Identification of Factor Scores by Regression with External Variables in Exploratory Factor Analysis

**DOI:** 10.1017/psy.2025.10025

**Published:** 2025-06-16

**Authors:** Naoto Yamashita

**Affiliations:** Faculty of Sociology, Kansai University, Suita, Osaka, Japan

**Keywords:** clustering, factor score indeterminacy, penalized estimation, regression

## Abstract

Factor score indeterminacy is a characteristic property of factor analysis (FA) models. This research introduces a novel procedure, regression-based factor score exploration (RFE), which uniquely determines factor scores and simultaneously estimates other parameters of the FA model. RFE uniquely determines factor scores by minimizing a loss function that balances FA and multivariate regression, regulated by a tuning parameter. Theoretical aspects of RFE, including the uniqueness of factor scores, the relationship between observed and latent variables, and rotational indeterminacy, are examined. Additionally, clustering-based factor exploration (CFE) is presented as a variant of RFE, derived by generalizing the penalty term to enable the clustering of factor scores. It is demonstrated that CFE creates cluster structures more accurately than the existing method. A simulation study shows that the proposed procedures accurately recover true parameter matrices even in the presence of error-contaminated data, with lower computational demand compared to existing methods. Real data examples illustrate that the proposed procedures provide interpretable results, demonstrating high relevance to the factor scores obtained by existing methods.

## 1 Introduction

Factor analysis (FA, Adachi, 2019; Bartholomew, 2011; Harman, 1976) is a method used to explain the variations among multiple observed variables by assuming unobserved variables called factors. Let 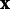 be a *p*-dimensional vector of random variables having mean vector 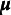 and covariance matrix 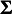 . The FA model with *r* common factors is formally defined as 
(1)

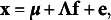

where 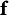 denotes an *r*-dimensional random vector representing common factor scores, which satisfies 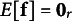 and 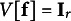 where 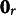 and 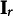 are the *r*-dimensional vector filled with zeros and the 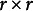 identity matrix, respectively, and 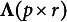 is the matrix of factor loadings. Furthermore, 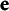 denotes a *p*-dimensional random vector of unique factor scores, which satisfies 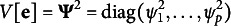 . This assumption indicates that 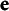 is decomposed as 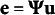 where 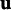 is a *p*-dimensional random vector satisfying 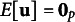 and 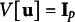 . The matrix 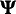 is assumed to be positive-semidefinite; 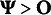 . 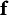 and 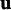 are assumed to be orthogonal: 
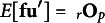
 where 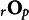 denotes 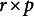 matrix filled with zeros. The unknown parameters in the model (1) are estimated by minimizing the discrepancy between the sample covariance matrix 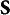 and 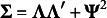 over 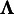 and 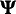 . For example, in maximum likelihood estimation, the negative log-likelihood function under the normality assumption on 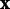 is defined as 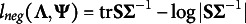 which is minimized to estimate 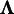 and 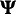 (Jöreskog, 1967; Lawley, 1940).

Alternative formulations of FA have been proposed by several authors (de Leeuw, 2004; Sočan, 2003; Stegeman, 2016). Among these procedures, matrix decomposition FA (MDFA; Adachi, 2022; Adachi & Trendafilov, 2018) and its theoretical and empirical properties have been investigated in recent years. The distinguishing property of MDFA is that it fits the FA model directly to the data matrix; thus, it is not formulated as a covariance fitting problem. To present the formulation of MDFA, let 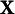 be the 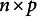 data matrix composed of horizontally stacked 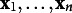 , where 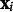 is the *p*-dimensional vector of the *i*th observation; 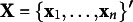 . Note that 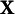 is assumed to be a column-centered matrix. Similarly, let 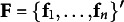 and 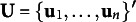 be the matrices of common and unique factor scores for *n* observations, respectively. MDFA minimizes the least squares loss function 
(2)

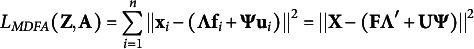

over the 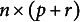 matrix of factor scores 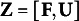 and the 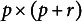 matrix 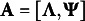 . The loss function in (2) evaluates the loss of fitness of the model part 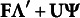 against the data matrix 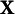 . The constraint on 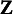 is noted as 
(3)

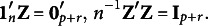



Factor indeterminacy is a characteristic property of FA (Maraun, 1996; Steiger, 1979, 1996), which causes the standard and unique factor scores, 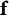 and 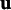 , to be not uniquely determined for all observations. Factor scores are also indeterminate in MDFA. Let the singular value decomposition of 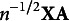 be defined as 
(4)

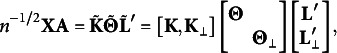

where 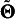 is a 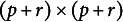 diagonal matrix of the singular values of 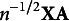 in descending order, and 
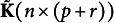
 and 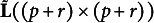 are matrices of the corresponding left and right singular vectors, respectively. Furthermore, the first *p* columns of 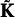 and 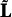 are denoted as 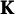 and 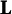 , respectively, and 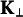 and 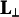 are matrices of the remaining columns. 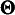 is a 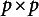 diagonal matrix of 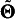 ’s first *p* diagonal elements, and 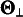 is a diagonal matrix of the remaining elements. The optimal 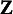 which minimizes (2) is given by 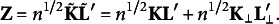 This suggests that the matrix of the optimal factor scores comprises two parts: 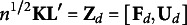 and 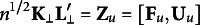 . Here, we have 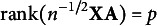 when 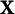 is a full-column-rank matrix where 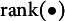 denotes rank of a matrix. The *r* smallest singular values of 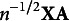 are zero, and 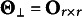 . Therefore, 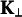 and 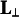 are not uniquely determined as long as they satisfy 
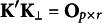
 and 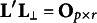 . 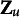 is not unique, indicating that the factor score matrix 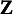 in MDFA is not uniquely determined. Note that 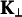 is not necessary to be column centered, while 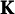 is column centered as long as 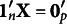 , and therefore 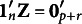 may not be satisfied in MDFA. MDFA explicitly separates the unique and non-unique parts of factor scores, 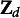 and 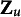 , allowing further consideration of factor score indeterminacy, unlike standard FA. Thus, we address this issue within the MDFA framework.

In this study, we propose a new method for identifying factor scores in the framework of MDFA. The proposed method is as an alternative to the existing procedure detailed in the next section, addressing its problems. Moreover, the proposed method proved to determine common and unique factor scores uniquely. The proposed method can be generalized to include various procedures dealing with latent variables.

To enhance the accessibility of our proposed methods, we have developed the R package mdfaScoreIden, available at https://github.com/nyamashita/mdfaScoreIden. This package includes the procedures introduced in this article.

The article is structured as follows. The next section reviews existing methods for specifying factor scores. The third section defines the proposed method, its optimization algorithm, and its properties, including the uniqueness of estimated scores. The fourth section presents a general formulation with special cases. The fifth and sixth sections report simulation studies and real data examples, respectively. The final section discusses conclusions and future research directions.

## 2 Existing procedures

The current section introduces two existing procedures to estimate or specify factor scores and addresses their limitations, as a motivation for the present study.

Although factor scores in FA are not uniquely determined, individual scores are often of interest in applied research. For example, Sergi et al. (2021) examined emotional intelligence, anxiety, and depression by regressing factor scores by FA, as did Gass (1996) and Zammuner (1998). To meet such needs, various post-hoc estimation methods exist, including regression, Bartlett’s (Bartlett, 1937), and Anderson & Rubin’s (Anderson & Rubin, 1956) methods. Bartlett’s method estimates 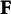 by minimizing 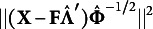 using parameter estimates 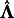 , 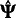 , and the rotated factor correlation matrix 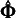 . The estimated factor scores, 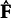 , are given by 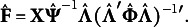 as a linear combination of observed variables in 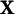 .

In MDFA, Uno et al. (2019) proposed clustered common factor exploration (CCFE) as a pioneering method for resolving factor score indeterminacy. CCFE assumes that individuals are classified into a few clusters, and those in the same cluster share similar common factor scores” (Uno et al., 2019), a desirable property for 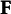 . Based on this assumption, the indeterminate part of 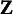 , 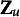 , is identified as follows.

First, by applying MDFA, the uniquely determined part of 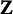 is computed as a linear combination of the *p* observed variables in 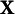 . Next, let 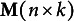 be an unknown membership matrix with elements 0 or 1 and row sums equal to 1, and 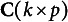 be an unknown cluster centroid matrix. Then, 
(5)

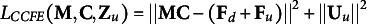

is minimized to determine 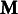 , 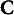 , and 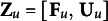 , where 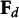 is the matrix of uniquely determined common factor scores. The minimization of 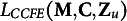 uniquely determines 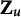 , as well as 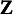 .

CCFE effectively identifies well-clustered common factor scores but has two limitations. First, classifying individuals into clusters based on factor scores is rarely ideal. In FA, it is often more meaningful to compare scores by demographics like gender or age rather than by estimated clusters. Factor scores numerically represent construct tendencies, such as intelligence, allowing comparisons across groups to reveal meaningful differences. For example, Weisberg et al. (2011) analyzes personality traits by comparing Big5 factor scores by gender, while Hughes et al. (2021) examines socioeconomic indicators. These studies relate factor scores to external criteria, which CCFE does not. As a result, CCFE clusters may not align with demographic groups, making interpretation difficult. Recent advances in exploratory FA using an additional set of variables are found in Carrioza et al. (2019) to improve interpretability of extracted factors.

**Figure 1:**
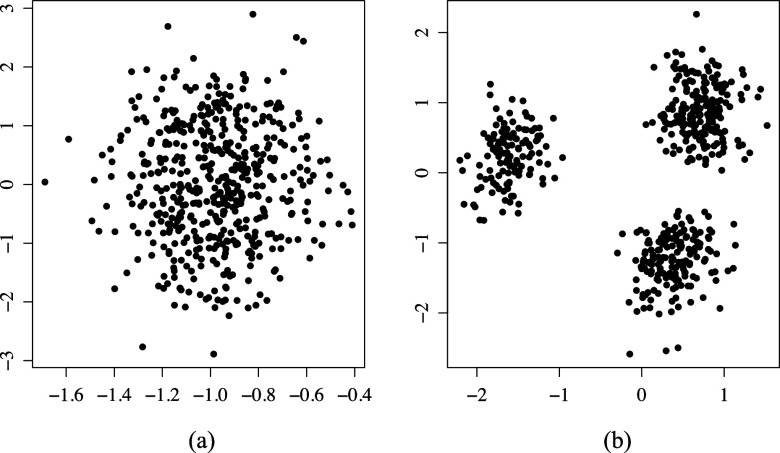
Result of CCFE applied to an artificial data having no cluster structure; (a) true common factor scores and (b) common factor scores estimated by CCFE.

The second problem is that CCFE creates clustered common factor scores, even when the scores have no cluster structure. To demonstrate this issue, the CCFE algorithm with three clusters was specifically applied to a 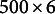 data matrix 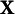 , which was synthesized in the following manner. 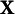 was synthesized as 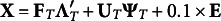 where 
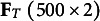
 and 
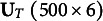
 are randomly-generated matrices of factor scores satisfying 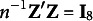 , with 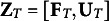 . 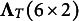 and 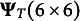 are defined as 
(6)

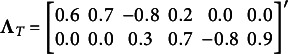

and 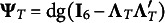 , where 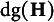 stands for the diagonal matrix substituting the off-diagonal elements of the square matrix 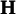 with zeros. 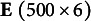 is a matrix of residuals filled with randomly generated elements drawn from the uniform distribution 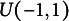 . Figure 1a,b displays scatter plots of true and estimated factor scores using CCFE.

The figure on the left indicates that the common factor scores exhibit no cluster structure, which is used in the data matrix 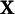 ’s generation process. However, the estimated factor scores on the right exhibit a three-cluster structure, which is unreasonable. The cluster structure created here by CCFE can be considered as illusionary because it does not exist in the data generation process. Regrettably, CCFE is the only method for determining factor scores in MDFA. Given the critical issues highlighted here, developing an alternative to CCFE is imperative.

## 3 Proposed method

This section first presents the mathematical formulation of the proposed method, followed by the iterative algorithm for estimating the unknown parameters. Subsequent subsections discuss the proposed method’s theoretical properties. The specification of the tuning parameter involved in the proposed method is also addressed.

### 3.1 Formulation

We propose a new procedure for specifying the factor scores, called regression-based factor score exploration (RFE). RFE is formulated as the minimization of the least-squares loss function 
(7)

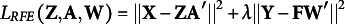

over the parameter matrices under the constraint on 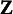 in (3). The first two parameter matrices, 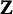 and 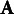 , match those in MDFA. In contrast, the newly introduced 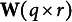 represents regression coefficients for *q* external criteria on *r* common factors. 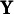 , the 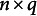 matrix of column-centered external criteria, is included. The first term in (7) is the MDFA’s loss, while the second represents multivariate regression loss, where 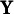 is explained by 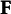 , with a predefined positive tuning parameter 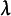 . Unlike post-hoc factor score estimation, RFE jointly estimates all parameter matrices including uniquely determined factor scores.

### 3.2 Algorithm

The closed-form solution for minimizing (7) does not exist, and the parameter matrices are estimated by the following iterative algorithm starting from their initial values.

To derive the algorithm, we first find the optimal 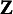 that minimizes the loss function in (7). First, the two block matrices 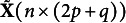 and 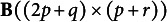 are defined as 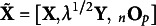 and 
(8)

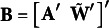

respectively, where 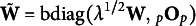 is a 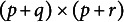 matrix, and 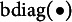 denotes a block diagonal matrix of the arguments with appropriate dimensions. Using these, equation (7) can be rewritten as 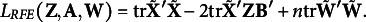 Therefore, minimizing equation (7) with respect to 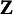 is equivalent to maximizing 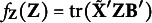 under (3). Furthermore, the maximization of 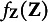 is achieved as 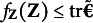 using the singular value decomposition 
(9)

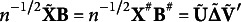

following ten Berge (1993), with 
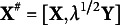
 and 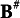 being the matrix omitting the last *p* rows of 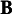 . 
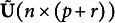
 and 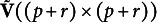 are matrices with the corresponding left and right singular vectors, respectively. 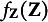 is maximized when 
(10)

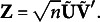

When 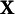 and 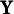 are column-centered matrices, 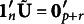 , so it is easy to verify that the 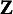 in equation (10) satisfies (3). It will be shown that the 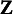 in equation (10) is uniquely determined.

Next, we seek 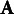 that minimizes equation (7). Following Adachi & Trendafilov (2018), the optimal 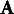 is given by 
(11)

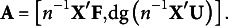

Finally, the 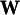 that minimizes the loss function (7) is obtained as 
(12)

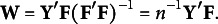

The derivation of the update formulae are presented in Supplementary Material 1.

Based on the above discussion, the iterative algorithm for parameter estimation in RFE can be summarized as follows:

1. Assign appropriate initial values to 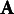 and 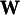 .
2. Update 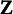 using equation (10).
3. Update 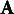 using equation (11).
4. Update 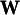 using equation (12).
5. If the decrease in the objective function between Steps 2–4 updates is less than 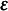 , terminate the algorithm. Otherwise, return to Step 2.

The function value is guaranteed to converge as discussed in Supplementary Material 1. Note that random initialization is necessary, and the algorithm will be started from *M* different initial values. The solution that provides the smallest function value will be adopted as the final solution.

The number of common factors *r* and the tuning parameter 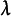 must be predefined, with 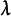 detailed in the next section. Given 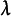 , *r* can be determined using standard methods like parallel analysis (Humphreys & Montanelli, 1975).

### 3.3 Properties

This subsection presents five distinctive properties that help emphasize the differences and similarities between the proposed method and existing procedures.

#### Uniqueness of factor scores

Theorem 1. The factor score matrix 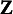 defined in (10) is uniquely determined when the following conditions hold: 
(13)

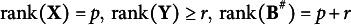

and 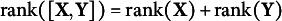 , indicating that columns of 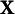 and 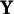 are linearly independent.

The proof is found in Supplementary Material 1. Among the conditions (13) necessary to uniquely determine 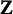 , the first conditions are generally satisfied in FA, as well as the third condition also holds considering (11) and (12). In contrast, the second condition and the linear independence of 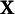 and 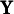 introduce a requirement for external criteria in RFE. Specifically, to ensure the unique determination of 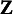 , the number of external criteria must be at least equal to the number of factors when 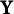 is a full-column-rank matrix.

Here, denote the parameter estimates obtained by RFE as 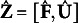 , 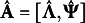 , and 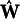 . Based on the uniqueness of 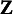 discussed above, the data matrix 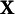 can be uniquely decomposed as 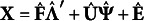 , where 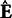 is the 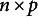 . Therefore, RFE allows to uniquely compute individual-wise residual by 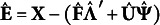 , which indicates the degree of incompliance of the observed data to FA model.

#### Relationship between the observed and latent variables

Theorem 2. The factor score matrix 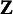 obtained by (10) is a linear combination of *p* and *q* observed variables in 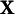 and 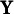 , respectively.

See Supplementary Material 2 for the proof. The theorem indicates that the column space of the factor score matrix estimated by RFE, 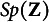 , is included in the column space of 
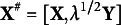
 , 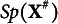 ; 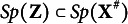 . This indicates that the factor score matrix obtained by RFE fundamentally differs from the factor scores obtained by standard FA followed by post-hoc estimation methods, which are represented as linear combinations of the observed variables 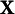 that are the subject of FA. However, the factor scores obtained by RFE cannot be represented in this way, and they are represented as linear combinations of 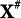 , which includes the external criteria used in RFE.

#### Rotational indeterminacy

Equation (7) can be rewritten using the block diagonal matrix 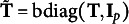 , where 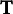 is an 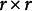 non-singular matrix satisfying 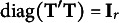 , as 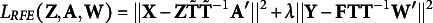 . From this, it can be seen that the parameter matrices 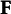 , 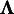 , and 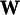 in RFE are indeterminate with respect to nonsingular transformations by 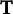 . 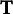 can be specified by using the oblique rotational procedure, and see Supplementary Material 3 for details of the specification.

#### RFE as a penalized FA procedure

As previously mentioned, the balance between the first and second terms in the overall objective function, or the extent to which the FA estimates are influenced by the regression represented by the second term, is controlled by the tuning parameter 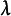 . Here, the second term in equation (7) can be expressed as 
(14)

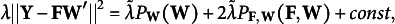

where 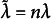 , 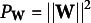 , and 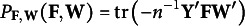 , and 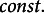 represents a constant term not related to 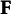 and 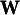 . Also, 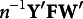 represents the covariance matrix of the *q* external criteria and the *q* composite variables weighted by 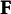 and 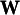 , considering 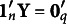 and 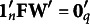 . From the above discussion, the objective function of RFE can be expressed as 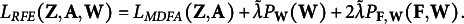 This indicates that RFE represents penalized MDFA, where penalty terms are added to various parameters of FA. The first penalty term 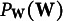 is an 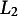 penalty term on 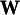 . The second penalty term 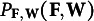 acts to increase the sum of the column covariances of 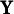 and 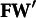 , thereby forming 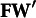 proportional to 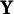 .

Several methods for penalized estimation of parameter matrices in FA have been proposed (Choi et al., 2011; Hirose & Terada, 2023; Hirose & Yamamoto, 2015). These procedures aim to simplify 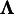 , which plays a central role in interpreting the FA’s results. In contrast, the RFE proposed in this study introduces penalized estimation of parameter matrices to uniquely determine factor scores by removing the indeterminacy of factor scores.

### Specification of 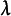


3.4

When executing the RFE algorithm, it is necessary to set the value of 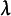 , which controls the balance between the first and second terms of the equation (7). The article recommends a semi-automatic procedure for selecting 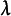 within a predefined range, as detailed in Supplementary Material 5. Alternatively, a small fixed value such as 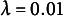 can be used, as supported by the following observations. Theorem 1 shows that 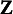 is uniquely determined under condition (13) when 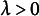 . Moreover, the simulation in the next section indicates that the RFE solution remains accurate and stable for small 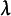 . Another simulation, reported in Supplementary Material 5, confirms that the semi-automatic procedure consistently selects 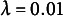 as optimal, demonstrating the effectiveness of using a small 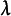 .

## 4 Related procedures and generalization

The loss function in (7) can be generalized by considering not only the regression of 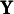 on 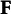 but also the reverse regression: 
(15)

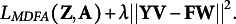

In this section, we demonstrate that by setting penalty terms on the parameter matrices added to the objective function of FA in various ways, we can endow the parameter matrices in FA with useful properties.

By setting the matrices other than 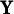 in the second term of (15), we can represent RFE, where 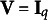 and 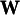 is treated as a free parameter.

Next, by setting 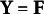 and treating it as a free parameter, while also setting 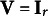 , equation (15) corresponds to the objective function of MDSEM by Yamashita (2024). MDSEM is a modeling framework capable of handling various models, including regressions among latent variables, which works as a structural equation modeling (Bentler, 1980; Mulaik, 1986). Note that the factor scores in MDSEM are not uniquely determined as proved in Yamashita (2024).

Another procedure is obtained by letting 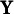 be the 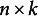 membership matrix 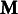 , and 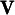 be the 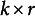 centroid matrix 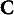 , with 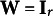 . This method formulates the second term as the objective function of *K*-means clustering analysis, which classifies the common factor scores 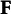 into *k* clusters: 
(16)

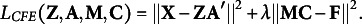

Thus, similar to CCFE by Uno et al. (2019), the obtained factor score matrix is expected to form *k* clusters. Hereafter, we will refer to this method as clustering-based factor exploration (CFE). The parameter estimation algorithm in CFE is detailed in Supplementary Material 5.

**Figure 2:**
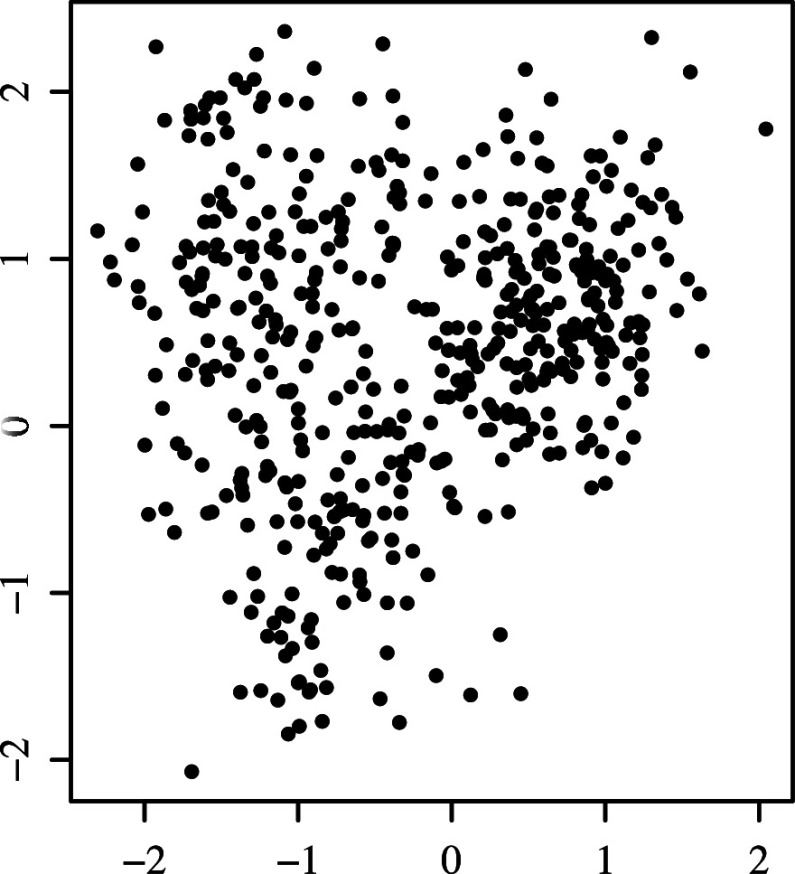
Factor scores estimated by CFE are applied to artificial data with no cluster structure.

A key limitation of CCFE is that it can create artificial cluster structures even when no cluster structure exists in the common factor scores. In contrast, CFE avoids this issue, as shown in Figure 2, where the cluster boundaries appear less distinct than in CCFE’s results. This difference is further reflected in the within-cluster variance and the distance between cluster centroids. For CFE, the minimum within-cluster variance and maximum between-cluster distance are 0.177 and 2.008, respectively, compared to 0.056 and 2.494 in CCFE. The clusters estimated by CCFE exhibit smaller within-cluster variance and more widely separated centroids than those obtained by CFE, indicating that CFE’s clusters are less distinct. This occurs because CFE balances FA and clustering through its penalization term in the loss function (16). Specifically, the tuning parameter was set to 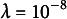 , as determined by the procedure in Supplementary Material 4, suggesting that clustering plays a minimal role in (16).

## 5 Numerical simulation

A numerical experiment was conducted to evaluate the effectiveness of the proposed method in accurately reproducing the true parameter matrices. This section reports the design and results of the experiment.

### 5.1 Design

The artificial data matrices 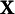 and 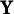 , to which the RFE proposed in the article is applied, were generated using the following procedure. First, the true factor score matrix 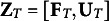 was randomly generated to satisfy (3). Specifically, an 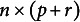 matrix 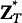 of the same size as 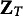 was generated with elements randomly drown from a uniform distribution 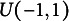 . Then, 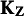 , the left singular vector matrix of the column-standardized 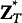 , was obtained as 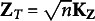 . Next, 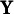 was constructed as 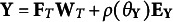 . Here, 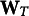 is the true value of 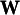 , which is set as 
(17)

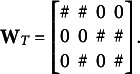

The matrix’s non-zero elements noted as # were generated from a uniform distribution 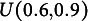 , with their signs randomly determined. Additionally, 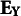 is an 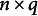 error matrix with elements generated from the standard normal distribution 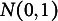 . The function 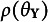 adjusts the relative size of the error matrix, with 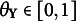 representing the variance explained, defined in Yamashita & Mayekawa (2015). Similarly, using the 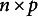 error matrix 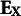 whose elements were generated from 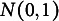 , 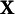 was generated as 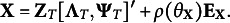 Here, 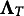 is the factor loading matrix defined as 
(18)

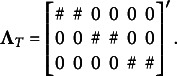

The non-zero elements were randomly generated from a uniform distribution 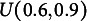 , with their signs randomly chosen. The true value of the uniquenesses’ square root 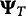 was set as 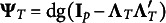 .

Three levels of error in the data, 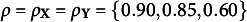 , were considered. For each level, 100 pairs of 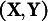 were generated using the above procedure with 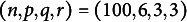 For each pair of data matrices, RFE was executed with six different values of 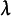 satisfying 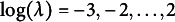 , and the estimated parameter matrices 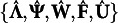 were obtained.

The deviation of the estimated parameter matrices from their true values was evaluated using the root mean square error of approximation (RMSEA) for any matrix 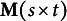 and its true counterpart 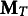 : 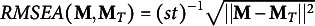 . The accuracy of the true values’ recovery for 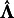 , 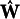 , 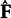 , and 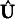 was evaluated using 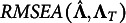 , 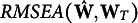 , 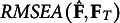 , and 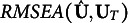 , respectively. For evaluating the recovery of 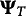 , the uniquenesses calculated from the estimates and their true values were compared using 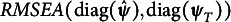 . 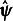 and 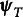 denote the vectors of the diagonal elements of 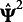 and 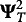 , respectively, which indicate the vectors of uniqueness.

The frequency of local solutions in each application of RFE was evaluated as follows. Let 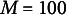 denote the number of initial values in RFE. After executing the algorithm, let the function values of equation (7) obtained post-convergence be 
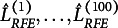
 . The minimum function value is 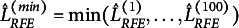 . The number of *j* satisfying 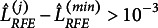 was counted as the frequency of local solutions, and this value divided by *M* was taken as the proportion of local solutions.

For comparison, we estimate maximum likelihood FA and Anderson & Rubin’s factor scores. RMSEA is computed for loadings, uniqueness, and common factor scores, but unique factor scores and 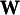 are unavailable in the existing method.

### 5.2 Result

Table 1 shows the median and standard deviation of RMSEA for the five parameter matrices for 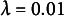 . The whole result is shown in Supplementary Material 6. For all parameter matrices, even in the case with the smallest variance explained rate, the median RMSEA is mostly below 0.1 with a small variance. This result suggests that the true parameter matrices recovered better than FA and Anderson & Rubin’s factor score. Additionally, the recovery accuracy of the parameter matrices does not change significantly with different values of 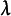 . This indicates that RFE is relatively robust to the value of 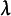 in terms of recovery accuracy of the parameter matrices, which is practically useful. The median and standard deviation of the frequency of local solutions are presented. Uno et al. (2019) empirically demonstrated that CCFE frequently encounters local solutions. In contrast, the proposed RFE method, as shown in the table, rarely encounters local solutions. Therefore, from the perspective of computational efficiency, RFE is superior to existing methods.

**Table 1:** Medians and standard deviations (s.d.) of 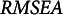 s of the estimated parameter matrices obtained by RFE in each condition and frequency of local minimum for 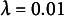

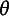		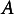	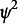	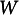	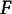	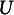	Local minimum
0.900	Median	0.014	0.038	0.036	0.019	0.025	0.000
	s.d.	0.003	0.011	0.007	0.002	0.002	0.076
0.850	Median	0.023	0.070	0.041	0.029	0.033	0.000
	s.d.	0.005	0.016	0.009	0.002	0.002	0.113
0.600	Median	0.032	0.098	0.052	0.037	0.039	0.000
	s.d.	0.006	0.021	0.014	0.003	0.002	0.160

## 6 Applications to real data

### 6.1 Big five inventory data

In the first application, we applied RFE and related existing methods to responses from a 25-item Big Five Inventory (Goldberg, 1999). A total of 2,800 participants completed the inventory as part of an online personality assessment project (Revelle et al., 2010). For this analysis, we used responses to ten items designed to serve as indicators of two latent factors: *Neuroticism* and *Openness*. Additionally, the dataset included respondents’ gender.

After removing cases with missing values, we obtained a 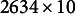 data matrix of item responses, denoted as 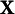 , for FA, and a 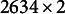 dummy-coded matrix for gender, denoted as 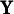 , to uniquely determine the factor scores. We applied RFE with two factors and compared it with maximum likelihood FA (MLFA). The regularization parameter for RFE was set to 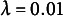 . Geomin rotation (Yates, 1987) was applied to the factor loading matrices obtained by both RFE and MLFA.

Table 2 compares the factor loadings and uniquenesses from RFE and MLFA, showing similar estimates. The simple structure observed in both methods supports interpreting the two factors as *Neuroticism* and *Openness*, consistent with MLFA, thereby supporting the validity of RFE.

**Table 2:** Factor loading matrices, uniquenesses (Uniq.), and inter-factor correlation matrices obtained by RFE and FA with maximum likelihood estimation (MLFA)

			RFE			MLFA	
		Neuroticism	Openness	Uniq.	Neuroticism	Openness	Uniq.
	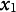	**0.786**	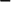 0.027	0.368	**0.808**	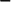 0.013	0.346
	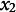	**0.778**	0.024	0.389	**0.800**	0.037	0.364
	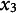	**0.749**	0.022	0.436	**0.725**	0.023	0.476
	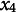	**0.593**	0.002	0.645	**0.563**	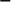 0.003	0.683
Factor loadings	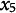	**0.512**	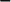 0.137	0.699	**0.498**	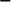 0.131	0.724
	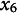	0.000	**0.547**	0.691	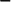 0.026	**0.553**	0.691
	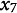	0.112	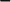 **0.451**	0.762	0.133	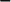 **0.445**	0.774
	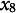	0.023	**0.629**	0.587	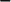 0.009	**0.638**	0.592
	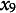	0.242	**0.326**	0.840	0.207	**0.322**	0.865
	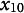	0.024	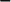 **0.527**	0.710	0.052	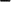 **0.519**	0.724
Inter-factor	Neuroticism	1.000	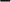 0.130		1.000	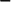 0.089	
correlation	Openness		1.000			1.000	

*Note*: The loadings larger than 0.4 in absolute are bolded.

Factor scores of MLFA were estimated post hoc using Bartlett’s method. The correlation coefficients between the Bartlett’s scores and those obtained via RFE were 0.942 and 0.917 for the two factors, respectively, indicating that RFE produces factor scores comparable to those from conventional FA with post hoc estimation. The critical difference between RFE and existing methods lies in the factor score distributions across demographic groups. RFE emphasizes score differences between individuals—particularly between genders—more strongly than methods such as Bartlett’s and those of ten Berge et al. (1999). Table 3 shows that gender differences in factor scores are more clearly captured with RFE = 0.95. These findings indicate that RFE not only yields unique factor scores but also enhances sensitivity to group-level differences that traditional methods tend to obscure.

**Table 3:** Factor score differences between genders (male 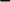 female) and 95% confidence intervals of Cohen’s (1988) *d* obtained by RFE, Bartlett’s, and ten Berge’s methods

	RFE		Bartlett’s method		Ten Berge’s method	
	Difference	Cohen’s *d*	Difference	Cohen’s *d*	Difference	Cohen’s *d*
Neuroticism	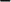 0.875	[ 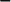 1.075, 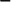 0.904]	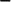 0.247	[ 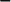 0.310, 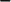 0.147]	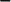 0.229	[ 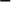 0.311, 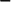 0.149]
Openness	0.854	[0.997, 1.170]	0.204	[0.085, 0.248]	0.169	[0.089, 0.252]

Weisberg et al. (2011) reported that among the Big Five factors, *Neuroticism* shows the largest gender difference, while differences in *Openness* are often masked by overlapping score distributions. Our method reveals such subtle differences more clearly, making it useful for studies comparing groups, such as Weisberg et al. (2011).

### 6.2 Job impression dataset

In the fourth section, we proposed CFE as a variant of RFE. In this subsection, we empirically demonstrate the usefulness of CFE by comparing it with the existing method CCFE by Uno et al. (2019).

**Figure 3:**
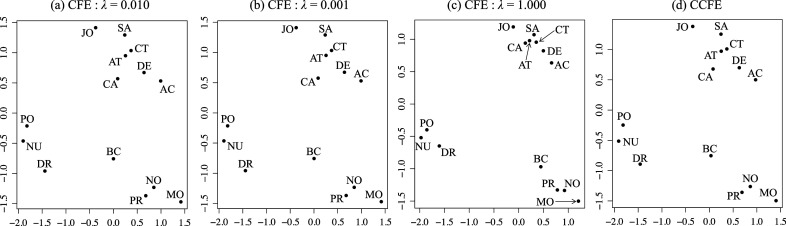
Factor scores estimated by CFE with three different 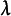 s (a-c) and CFE (d) applied to the job impression dataset. *Note*: In all figures, the horizontal and vertical axes of the figures stand for positive image and busyness, respectively. The occupations are abbreviated as follows: monk (MO) bank clerk (BC), comic artist (CT), designer (DE), nurse (NU), professor (PR), doctor (DR), police officer (PO), journalist (JO), sailor (SA), athlete (AT), novelist (NO), actor (AC), and cabin attendant (CA)

We applied CFE and CCFE to the occupational impression data (Committee for Guiding Psychological Experiments, 1985; Uno et al., 2019) collected in Japan. The occupational impression data consists of ratings of fourteen occupations listed in Figure 3 using six adjectives: *Useful*, *Good*, *Firm*, *Quick*, *Noisy*, and *Busy*. The respondents were asked to evaluate the extent to which their impression of each occupation applies to each adjective and their responses were averaged over the respondents; therefore, the data matrix 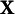 used for the analysis is a 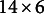 matrix. We applied CFE and CCFE to the standardized data matrix 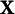 with the number of factors and clusters set to 2 and 3, respectively. These settings for the number of factors and clusters were the same as those used by Uno et al. (2019) for comparison. In CFE, the value of 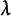 was set to 0.01. Additionally, to identify the orthogonal matrix 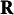 which makes the factor scores obtained by CFE and CCFE comparable, 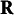 was determined to minimize 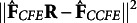 where 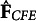 and 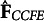 represent the estimated common factor score matrices obtained by CFE and CCFE, respectively. The optimal 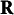 can be obtained by the orthogonal Procrustes rotation of 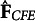 with the target matrix 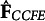 (Gower & Dijksterhuis, 2004). The CFE algorithm converged successfully in the current application. Standard FA, in contrast, can produce improper solutions with small samples in 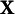 , yielding near-zero uniqueness ( 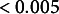 ), close to negative variance. MDFA, CFE, and CCFE mitigate this issue, as discussed in the final section.

The estimated factor loading matrices and uniquenesses are shown in Supplementary Material 9. The two factor loading matrices exhibit very similar simple structures, with the first factor showing large positive loadings on *Useful*, *Good*, and *Firm*, which can be interpreted as a positive image of the occupation. The second factor shows high positive loadings on *Quick*, *Noisy*, and *Busy*, which can be interpreted as representing the busyness of the occupation. Figure 3 shows scatter plots of the estimated common factor scores obtained by RFE using three different values of 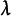 , including 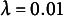 , and the factor scores obtained by CCFE. The plots of the factor scores obtained by RFE in Figure 3a and those obtained by CCFE in Figure 3d are very similar, demonstrating that CFE provides consistent factor score estimates with the existing method. The correlation coefficients between the factor scores obtained by CFE and CCFE were very high, 0.999 for the first factor and 0.990 for the second factor. Furthermore, the individual memberships obtained by both methods were completely consistent. The first cluster is composed of *police officer*, *nurse*, and *doctor*, the second cluster is composed of *bank clerk*, *novelist*, *professor*, and *monk*. The remaining seven occupations are in the third cluster. Figure 3 shows that comic artists and athletes are closely positioned within the same cluster, reflecting their shared cultural image as “good” and “busy” professions in Japan, where comic artists are also seen as engaging in demanding work (Ristola, 2016).

Figure 3b,d shows the plots of the factor scores obtained by applying CFE with different 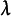 values. The factor scores for 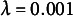 are similar to those of CFE with 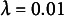 and CCFE, but the factor scores for 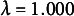 considerably differ from the others. The factor scores obtained with 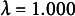 are positioned closer to the cluster centers for each individual, resulting in larger within-cluster variance than the other results. This indicates that the proposed CFE method allows adjusting the degree of cluster cohesion of the factor scores by tuning the value of 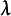 . Moreover, since the individual membership information output by CFE perfectly matches that of CCFE for all 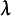 values, it can be inferred that the cluster structures of individuals extracted by both methods are consistent with each other.

## 7 Concluding remarks

Factor score indeterminacy is a fundamental property of FA, but scores often need specification for further analysis. The proposed method RFE addresses this by integrating score estimation into FA using external criteria. Further, it generalizes to methods like CFE and MDSEM. This study focuses on CFE, which extends RFE’s penalty to enable clustering while avoiding artificial structures, improving on Uno et al. (2019).

Even though MDFA is less popular than standard FA, it offers several advantages. First, unlike standard FA, which treats factor scores as random variables, MDFA estimates them directly as model parameters (Adachi, 2019), making factor score specification reasonable. Second, MDFA avoids improper solutions where uniqueness is negative, as 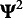 ensures non-negativity. Third, MDFA allows for observation-specific residual evaluation without additional computations, unlike standard FA (Adachi, 2022). Moreover, MDFA has proven to have statistical properties, including consistency (Terada, 2024), making it a promising FA procedure.

The key difference between RFE and CCFE is their approach to factor score estimation. RFE uses penalized FA to uniquely estimate all parameters, including factor scores, making 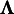 and 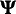 differ from standard FA and aligning RFE more with multi-task learning than strict score identification. CCFE, however, resolves factor score indeterminacy while keeping 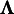 and 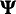 identical to standard FA. Simulations show RFE estimates true parameters well when 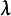 is small, making its penalization impact minimal and unlikely to affect interpretation.

The unique determination of 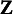 allows 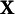 to be decomposed as 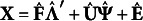 , where the first term represents common factor scores, the second unique factor scores, and the third unexplained variance shown. This decomposition enables individual analysis without post-hoc calculations required in standard FA. However, interpreting unique factors requires more discussion, as they are rarely considered in FA.

Semiparametric and regularized FA methods perform well under the assumptions on loading homogeneity and technical identification constraints (Guo & Li, 2022; Li et al., 2018). This study instead highlights the importance of individual-level factor scores, which are often of primary interest in psychological research. Such applications require scores to be uniquely specified and interpretable. The proposed method addresses this need by resolving indeterminacy through regularization with external information. To the best of our knowledge, no existing method has provided a unified approach that ensures both uniqueness and interpretability of individual scores. Our method uniquely determines factor scores in a way that reflects meaningful patterns, such as demographic differences, which are difficult to capture with existing approaches.

Despite their advantages, the proposed methods have limitations. A key issue is the reliance on well-chosen external criteria in RFE; if poorly selected, factor scores may be ambiguous or less interpretable. Proper 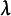 selection in RFE and CFE is also critical, as improper tuning can distort clustering or score determination. While sufficiently small 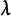 works well, further research is needed for broader applicability.

Future research directions include extending RFE and CFE to more complex models, such as hierarchical or multi-level FA and structural equation modeling, to broaden their applicability. Additionally, exploring integrating alternative penalty functions within the RFE framework could improve parameter estimation and interpretability.

In conclusion, RFE and CFE represent significant advancements in FA, offering unique solutions to the limitations of traditional methods. Their robust performance in simulations and real data applications underscores their practical relevance and potential for broad application across various research domains. These methods provide a solid foundation for future developments in FA and related fields, promising enhanced interpretability and flexibility in understanding complex data structures.

## Supporting information

Yamashita supplementary material Yamashita supplementary material

## Data Availability

R package mdfaScoreIden is available via GitHub. Please visit https://github.com/nyamashita/mdfaScoreIden for detail.

## Supplementary material

The supplementary material for this article can be found at https://doi.org/10.1017/psy.2025.10025.
