# Supplementary material for: Identification of Factor Scores by Regression with External Variables in Exploratory Factor Analysis
Source: Psychometrika. 2025 Jun 16;90(3):1097–110. doi: 10.1017/psy.2025.10025 (PMC12483718; doi:10.1017/psy.2025.10025)
Supplement: Yamashita supplementary material [file S0033312325100252sup001.pdf]

Supplementary Material 1

The update formulae for $\mathbf{A}$ and $\mathbf{W}$, given in equations (11) and (12), are derived as follows.

First, consider minimizing the RFE loss function $L_{RFE}(\mathbf{Z}, \mathbf{A}, \mathbf{W})$ with respect to $\mathbf{A}$, given fixed $\mathbf{Z}$ and $\mathbf{W}$. This is equivalent to minimizing the function $f_{\mathbf{A}}(\mathbf{A}) = ||\mathbf{X} - \mathbf{Z}\mathbf{A}'||^2$. Taking into account the constraint that $\mathbf{Z}$ satisfies equation (3), the normal equation for this minimization problem is given by

$$\frac{\partial f_{\mathbf{A}}(\mathbf{A})}{\partial \mathbf{A}} = 2\mathbf{A}\mathbf{Z}'\mathbf{Z} - 2\mathbf{X}'\mathbf{Z} = {}_p\mathbf{O}_{p+r} \tag{S1.1}$$

$$\mathbf{A} = \frac{1}{n}\mathbf{X}'\mathbf{Z}. \tag{S1.2}$$

Here, noting that $\mathbf{A} = [\mathbf{\Lambda}, \mathbf{\Psi}]$ with $\mathbf{\Psi}$ being a diagonal matrix, the update formula (11) follows accordingly.

It is important to ensure that the components $\mathbf{\Lambda}$ and $\mathbf{\Psi}$ of $\mathbf{A}$ have full column rank, which guarantees that $\text{rank}(\mathbf{B}^{\#}) = p + r$ as assumed in the proof of Theorem 1 (see Supplementary Material 2). This condition is satisfied if both $\mathbf{F}$ and $\mathbf{U}$ have full column rank, which is ensured by the second condition in (3).

Next, consider minimizing $L_{RFE}(\mathbf{Z}, \mathbf{A}, \mathbf{W})$ with respect to $\mathbf{W}$, holding $\mathbf{Z}$ and $\mathbf{A}$ fixed. This is equivalent to minimizing $f_{\mathbf{W}}(\mathbf{W}) = ||\mathbf{Y} - \mathbf{F}\mathbf{W}'||^2$. Solving the corresponding normal equation yields

$$\frac{\partial f_{\mathbf{W}}(\mathbf{W})}{\partial \mathbf{W}} = 2\mathbf{W}\mathbf{F}'\mathbf{F} - 2\mathbf{Y}'\mathbf{F} = {}_p\mathbf{O}_{p+r}. \tag{S1.3}$$

Assuming $n^{-1}\mathbf{F}^\top\mathbf{F} = \mathbf{I}_r$, we obtain the update formula (12).

Let $\{(\mathbf{Z}^{(t)}, \mathbf{A}^{(t)}, \mathbf{W}^{(t)})\}_{t=1}^{\infty}$ denote the sequence of parameter estimates generated by the proposed alternating minimization algorithm. At each iteration t, the update of each parameter matrix is performed via a minimization of the objective function L_{RFE} with respect to that matrix, holding the others fixed. By construction, this yields a non-increasing sequence of objective values:

$$L_{\text{RFE}}(\mathbf{Z}^{(t+1)}, \mathbf{A}^{(t+1)}, \mathbf{W}^{(t+1)}) \leq L_{\text{RFE}}(\mathbf{Z}^{(t)}, \mathbf{A}^{(t)}, \mathbf{W}^{(t)}), \quad \forall t \in N. \tag{S1.4}$$

Moreover, the objective function is bounded below by zero:

$$L_{\mathrm{RFE}}(\mathbf{Z}, \mathbf{A}, \mathbf{W}) \geq 0, \quad \forall\, \mathbf{Z}, \mathbf{A}, \mathbf{W}. \tag{S1.5}$$

It follows that $L_{\mathrm{RFE}}(\mathbf{Z}^{(t)}, \mathbf{A}^{(t)}, \mathbf{W}^{(t)})$ is a bounded monotonic sequence and hence convergent.

While the convergence of the parameter sequence $(\mathbf{Z}^{(t)}, \mathbf{A}^{(t)}, \mathbf{W}^{(t)})$ to a stationary point is not guaranteed in general due to the potential non-convexity of the objective function, the convergence of the function values is ensured under standard assumptions.

Supplementary Material 2

Theorem 1 is proved as follows.

Proof. Considering the definitions of $n \times (p+q)$ matrix $\mathbf{X}^{\#}$ and $(p+q) \times (p+r)$ matrix $\mathbf{B}^{\#}$, we have

$$\mathrm{rank}(n^{-1/2}\mathbf{X}^{\#}\mathbf{B}^{\#}) \leq \ min(n, p+q, p+r). \tag{S2.1}$$

Assuming that $\mathbf{X}$ is a full-column rank matrix and that the columns of $\mathbf{Y}$ are not dependent on those of $\mathbf{X}$, we have

$$\mathrm{rank}(n^{-1/2}\mathbf{X}^{\#}\mathbf{B}^{\#}) = \min(p + \mathrm{rank}(\mathbf{Y}), \mathrm{rank}(\mathbf{B}^{\#})) \tag{S2.2}$$

Therfore, when

$$\mathrm{rank}(\mathbf{Y}) \geq r \tag{S2.3}$$

and

$$\mathrm{rank}(\mathbf{B}^{\#}) = p + r \tag{S2.4}$$

the rank of $n^{-1/2}\mathbf{X}^{\#}\mathbf{B}^{\#}$ is equal to $p+r$, and all $p+r$ singular values of the matrix are greater than zero, while the r smallest singular values in (4) used in MDFA are equal to zero. All the singular vectors in $\tilde{\mathbf{U}}$ and $\tilde{\mathbf{V}}$ correspond to non-zero singular values and they are uniquely determined, which causes $\mathbf{Z}$ obtained by (10) is uniquely determined.

Supplementary Material 3

Theorem 2 is proved as follows.

Proof. Noting (9), we obtain

$$\sqrt{n}\tilde{\mathbf{U}} = \mathbf{X}^{\#}\mathbf{B}^{\#}\tilde{\mathbf{V}}\tilde{\mathbf{\Delta}}^{-1}. \tag{S3.1}$$

Substituting this into (10), we have

$$\mathbf{Z} = \mathbf{X}^{\#}\mathbf{B}^{\#}\tilde{\mathbf{V}}\tilde{\mathbf{\Delta}}^{-1}\tilde{\mathbf{V}}' \tag{S3.2}$$

This equation means that the factor score matrix $\mathbf{Z}$ is obtained by right-multiplying the weighted coefficient matrix $\mathbf{B}^{\#}\tilde{\mathbf{V}}\tilde{\mathbf{\Delta}}^{-1}\tilde{\mathbf{V}}'$ to $\mathbf{X}^{\#} = [\mathbf{X}, \lambda^{1/2}\mathbf{Y}]$, which completes the proof. □

Supplementary Material 4

In order to specify the nonsingular matrix $\mathbf{T}$ in RFE, several methods can be considered to eliminate this indeterminacy and uniquely determine $\mathbf{T}$, as listed below.

First, it is possible to use factor rotation methods to approximate $\mathbf{\Lambda}$ to a simple structure. Approximating $\mathbf{\Lambda}$ to a simple structure can be achieved using algorithms for oblique rotation widely used in standard FA, such as Promax rotation (Hendrickson & White, 1964) and Geomin rotation (Yates, 1987).

Second, it is also feasible to approximate $\mathbf{F}$ to a simple structure. For example, using criteria such as the Varimax criterion (Kaiser, 1958), which is maximized when $\mathbf{FT}$ has only one non-zero element in each row, can yield results similar to CCFE, which aims for the clustering of common factor scores.

Third, it is possible to approximate $\mathbf{W}$ to a simple structure. By considering oblique rotation for $\mathbf{W}$, $\mathbf{T}$ can be determined. As will be illustrated in later applications, $\mathbf{W}$ describes the relationship between common factor scores and external criteria, and simplifying this matrix can make it easier to understand the relationship between the two variables clearly.

Fourth, a method to find $\mathbf{T}$ that simplifies all parameter matrices simultaneously can be considered. For this purpose, let $S(\bullet)$ be a function that evaluates the complexity of any matrix. Then, $\mathbf{T}$ is specified as the one minimizing

$$l(\mathbf{T}) = S(\mathbf{FT}) + S(\mathbf{\Lambda}\mathbf{T}^{-1\prime}) + S(\mathbf{W}\mathbf{T}^{-1\prime}) \tag{S4.1}$$

Geomin criterion and CLF criterion (Jennrich, 2006) can be considered as functions for complexity evaluation. Similar methods have been proposed by Kiers (1998) and Yamashita (2024).

References

Hendrickson, A. E., & White, P. O. (1964). Promax: A quick method for rotation to oblique simple structure. *British journal of statistical psychology*, *17*(1), 65–70.

Jennrich, R. I. (2006). Rotation to simple loadings using component loss functions: The oblique case. *Psychometrika*, *71*, 173-191.

Kaiser, H. F. (1958). The varimax criterion for analytic rotation in factor analysis. *Psychometrika*, *23*, 187-200.

Kiers, H. A. L. (1998). Joint orthomax rotation of the core and component matrices resulting from three-mode principal components analysis. *Journal of Classification*, *15*, 245-263.

Yamashita, N. (2024). Exploratory Procedure for Component-Based Structural Equation Modeling for Simple Structure by Simultaneous Rotation. *Psychometrika*, *89*, 411–438.

Yates, A. (1987). *Multivariate exploratory data analysis: A perspective on exploratory factor analysis*. Suny Press.

Supplementary Material 5

The specification process of λ using Akaike Information Criterion (AIC, Akaike, 1974) is detailed below.

As previously mentioned, RFE can be considered a penalized FA with penalties applied to $\mathbf{F}$ and $\mathbf{W}$. Previous studies on penalized FA (Choi et al., 2011; Hirose & Terada, 2022; Hirose & Yamamoto, 2015) have adopted AIC to set the tuning parameters. Here, we consider following this method and selecting the value of λ that minimizes the AIC among the candidate values.

Let $\hat{\mathbf{\Lambda}}$ and $\hat{\mathbf{\Psi}}$ denote the estimators of $\mathbf{\Lambda}$ and $\mathbf{\Psi}$ obtained by the RFE algorithm. Furthermore, let $\hat{\mathbf{T}}$ represent the $\mathbf{T}$ obtained through rotation aimed at simplifying the structure of $\mathbf{\Lambda}$, utilizing the indeterminacy of rotation mentioned in the second section. Based on these, the covariance structure of the parameter matrices estimated by RFE is given by

$$\hat{\mathbf{\Sigma}}(\hat{\mathbf{\Lambda}}, \hat{\mathbf{\Psi}}) = \hat{\mathbf{\Lambda}}\hat{\mathbf{\Phi}}\hat{\mathbf{\Lambda}}' + \hat{\mathbf{\Psi}}^2 \tag{S5.1}$$

where $\hat{\mathbf{\Phi}} = \hat{\mathbf{T}}'\hat{\mathbf{T}}$. Using (S5.1), the corresponding log-likelihood is obtained as

$$\hat{l}(\hat{\mathbf{\Lambda}}, \hat{\mathbf{\Psi}}) = \log |\mathbf{S}_{\mathbf{X}}\hat{\mathbf{\Sigma}}(\hat{\mathbf{\Lambda}}, \hat{\mathbf{\Psi}})^{-1}| - \mathrm{tr}\mathbf{S}_{\mathbf{X}}\hat{\mathbf{\Sigma}}(\hat{\mathbf{\Lambda}}, \hat{\mathbf{\Psi}})^{-1} \tag{S5.2}$$

where $\mathbf{S}_{\mathbf{X}} = n^{-1}\mathbf{X}'\mathbf{X}$ is the sample covariance matrix of $\mathbf{X}$. Based on these results, the AIC for the parameter matrices obtained by RFE is given by

$$AIC(\lambda) = -2\hat{l}(\hat{\mathbf{\Lambda}}, \hat{\mathbf{\Psi}}) + 2d \tag{S5.3}$$

where d is the degrees of freedom of the FA model and is given by

$$d = p(r+1) - \frac{r(r-1)}{2}. \tag{S5.4}$$

The optimal λ can be selected as the one minimizing $AIC(\lambda)$ among candidates of values for λ.

The above result provides an semi-automated approach for selecting the optimal λ, which is beneficial for potential users of RFE. Given a set of candidate values for λ, such as $\lambda = 10^{-3.0}, 10^{-2.5}, \cdots, 10^{1.0}$, RFE is applied to the data matrix for each λ to compute the corresponding $AIC(\lambda)$ values. The optimal λ is determined as the one that minimizes $AIC(\lambda)$.

Furthermore, the simulation results in the next section demonstrate that estimation accuracy remains stable across different λ values. Notably, due to the L_2 penalty used in RFE, the estimation outcomes show limited sensitivity to small variations in λ. These findings indicate that incorrect results are unlikely in most cases, even if λ is not precisely specified.

As Theorem 1 indicates, factor scores are uniquely determined when $\lambda > 0$. Therefore, choosing a sufficiently small λ ensures identifiability of the factor scores, which is one of the main objectives of the proposed method. To demonstrate that a small λ performs well—and that this choice is consistent with selections made by an AIC/BIC-based semi-automatic procedure—we conducted the following numerical simulation. The data generation process and experimental design were identical to those described in the earlier Numerical Simulation section. For each dataset $\mathbf{X}$, we selected the optimal λ value using both AIC and BIC from among six candidate values, defined as $\log(\lambda) = -3, -2, \ldots, 2$.

The selected λ values are summarized in Table S5.1. Both AIC and BIC selected $\lambda = 0.01$ as optimal in nearly all cases, indicating that setting λ to a small value such as 0.01—even without a tuning procedure—performs well across a wide range of scenarios.

TABLE S5.1.
Modes and standard deviations (s.d.) of the optimal λs selected by *AIC* and *BIC*.

θ		*AIC*	*BIC*
0.900	mode	0.010	0.010
	s.d.	0.000	0.000
0.850	mode	0.010	0.010
	s.d.	0.000	0.000
0.600	mode	0.010	0.010
	s.d.	0.000	0.000

References

Akaike, H. (1974). A new look at the statistical model identification. *IEEE Transactions on Automatic Control*, *19*, 716–723.

Choi, J., Zou, H., & Oehlert, G. (2011). A penalized maximum likelihood approach to sparse factor analysis. *Statistical Interface*, *3*(4), 429–-436.

Hirose, K., & Terada, Y. (2023). Sparse and simple structure estimation via prenet penalization. *Psychometrika*, *88*, 1381–1406.

Hirose, K., & Yamamoto, M. (2015). Sparse estimation via nonconcave penalized likelihood in factor analysis model. *Statistics and Computing*, *25*, 863–875.

Supplementary Material 6

The estimates for the parameter matrices in CFE can be obtained by repeating the steps to minimize (16) for each parameter matrix under appropriate initial values until the decrease in the objective function converges. For the parameter matrix $\mathbf{A}$, we can obtain the $\mathbf{A}$ that minimizes (16) as in RFE using (11). The optimal $\mathbf{Z}$ is obtained by using the singular value decomposition of $\sqrt{n}\mathbf{X}^{\#}\mathbf{B}^{\#}$ with $\mathbf{Y}$ and $\mathbf{W}$ replaced by $\mathbf{MC}$ and $\mathbf{I}_r$ in (9), respectively. However, regarding this singular value decomposition, rank($\mathbf{MC}$) $\leq \min(k, r)$ ensures that all singular values are larger than zero and the factor scores are uniquely determined as long as $k \geq r + 1$ holds. Next, let m_{ij} denote the (i, j) element of $\mathbf{M}$ representing the membership of the i-th individual in cluster j. The m_{ij} that minimizes (16) is obtained as

$$m_{ij} = \begin{cases} 1 & (j = \text{argmin}_{l=1,\cdots,k}||\mathbf{f}_{(i)} - \mathbf{c}_{(l)}||^2) \\ 0 & (otherwise) \end{cases} . \tag{S6.1}$$

Here, $\mathbf{f}_{(i)}$ and $\mathbf{c}_{(l)}$ represent the i-th row vector of $\mathbf{F}$ and the l-th row vector of $\mathbf{C}$, respectively. Finally, the $\mathbf{C}$ that minimizes (16) is obtained as:

$$\mathbf{C} = (\mathbf{M}'\mathbf{M})^{-1}\mathbf{M}'\mathbf{F}. \tag{S6.2}$$

The inverse of $\mathbf{M}'\mathbf{M}$ is uniquely determined only when there are no empty clusters among the k clusters. Moreover, equation (16) can be rewritten using the block diagonal matrix $\tilde{\mathbf{R}} = \text{bdiag}(\mathbf{R}, \mathbf{I}_p)$, which includes the orthogonal matrix $\mathbf{R}(r \times r)$, as

$$L_{CFE}(\mathbf{Z}, \mathbf{A}, \mathbf{M}, \mathbf{C}) = ||\mathbf{X} - \mathbf{Z}\tilde{\mathbf{R}}\tilde{\mathbf{R}}'\mathbf{A}'||^2 + \lambda||\mathbf{MCR} - \mathbf{FR}||^2 = L_{CFE}(\mathbf{Z}\tilde{\mathbf{R}}, \mathbf{A}\tilde{\mathbf{R}}, \mathbf{M}, \mathbf{CR}). \tag{S6.3}$$

To determine $\mathbf{R}$, an appropriate orthogonal rotation algorithm can be used for $\mathbf{\Lambda}$, which can simplify the structure of $\mathbf{\Lambda}$.

Supplementary Material 7

The whole results of the simulation study are shown in the tables below.

Table S7.1.

Medians and standard deviations (s.d.) of frequency of local minimum occurred in each condition.

	$\log(\lambda)$	-3	-2	-1	0	1	2
θ							
0.900	median	0.000	0.000	0.000	0.000	0.000	0.000
	s.d.	0.184	0.076	0.098	0.089	0.000	0.000
0.850	median	0.000	0.000	0.000	0.000	0.000	0.000
	s.d.	0.203	0.113	0.113	0.030	0.000	0.000
0.600	median	0.075	0.000	0.000	0.000	0.000	0.000
	s.d.	0.213	0.160	0.162	0.000	0.000	0.000

Table S7.2.

Medians and standard deviations (s.d.) of $RMSEA$s of the estimated parameter matrices obtained by RFE and FA in each condition.

		$\log(\lambda)$	-3	-2	-1	0	1	2	FA
	θ								
**A**	0.900	median	0.021	0.014	0.012	0.014	0.015	0.015	0.030
		s.d.	0.006	0.003	0.003	0.002	0.002	0.006	0.007
	0.850	median	0.030	0.023	0.021	0.025	0.028	0.028	0.119
		s.d.	0.006	0.005	0.003	0.003	0.003	0.003	0.031
	0.600	median	0.040	0.032	0.029	0.035	0.038	0.039	0.040
		s.d.	0.007	0.006	0.004	0.004	0.004	0.005	0.004
$\boldsymbol{\psi}^2$	0.900	median	0.075	0.038	0.031	0.038	0.044	0.045	0.036
		s.d.	0.027	0.011	0.007	0.008	0.009	0.009	0.008
	0.850	median	0.099	0.070	0.067	0.088	0.099	0.100	0.136
		s.d.	0.026	0.016	0.013	0.012	0.013	0.013	0.029
	0.600	median	0.126	0.098	0.098	0.127	0.134	0.135	0.044
		s.d.	0.028	0.021	0.018	0.018	0.018	0.018	0.004
**W**	0.900	median	0.035	0.036	0.037	0.038	0.041	0.042	
		s.d.	0.006	0.007	0.007	0.007	0.008	0.010	
	0.850	median	0.043	0.041	0.043	0.055	0.070	0.071	
		s.d.	0.009	0.009	0.010	0.013	0.015	0.016	
	0.600	median	0.053	0.052	0.069	0.107	0.121	0.122	
		s.d.	0.013	0.014	0.018	0.024	0.031	0.032	
**F**	0.900	median	0.024	0.019	0.019	0.019	0.021	0.021	0.045
		s.d.	0.004	0.002	0.001	0.001	0.001	0.003	0.008
	0.850	median	0.033	0.029	0.029	0.031	0.033	0.033	0.155
		s.d.	0.004	0.002	0.002	0.002	0.002	0.002	0.032
	0.600	median	0.041	0.037	0.038	0.042	0.044	0.044	0.052
		s.d.	0.004	0.003	0.002	0.002	0.002	0.002	0.003
**U**	0.900	median	0.027	0.025	0.024	0.024	0.024	0.024	
		s.d.	0.002	0.002	0.002	0.002	0.002	0.002	
	0.850	median	0.035	0.033	0.033	0.033	0.033	0.033	
		s.d.	0.002	0.002	0.002	0.002	0.002	0.002	
	0.600	median	0.041	0.039	0.038	0.038	0.038	0.038	
		s.d.	0.002	0.002	0.002	0.002	0.002	0.002	

Supplementary Material 8

The estimated loading and uniqueness by CFE and CCFE in the second example are shown below.

Table S8.1.

Factor loading matrices and uniquenesses (Uniq.) estimated by CFE and CCFE applied to the job impression dataset. The loadings larger than 0.4 in absolute are bolded.

	CFE			CCFE		
	F1	F2	Uniq.	F1	F2	Uniq.
Useful	**0.842**	0.125	0.199	**0.874**	0.129	0.213
Good	**0.763**	0.013	0.338	**0.790**	0.014	0.367
Firm	**0.754**	0.131	0.337	**0.785**	0.135	0.359
Quick	0.062	**0.790**	0.293	0.067	**0.817**	0.321
Noisy	0.068	**0.898**	0.108	0.069	**0.938**	0.106
Busy	**0.404**	**0.670**	0.310	**0.420**	**0.694**	0.336
